# Supplementary material for: HPV Vaccine: An Effective but Underutilized Prevention Tool
Source: Int J Environ Res Public Health. 2025 Dec 10;22(12):1844. doi: 10.3390/ijerph22121844 (PMC12732589; doi:10.3390/ijerph22121844)
Supplement: Supplementary file 1 [file ijerph-22-01844-s001.zip › ijerph-3903575-supplementary.pdf]

International Journal of
Environmental Research and Public Health

MDPI

Table S1: STROBE Statement—Checklist of items that should be included in reports of ***cohort studies***

	Item No	Recommendation	Page No.	Relevant text from manuscript
**Title and abstract**	1	(*a*) Indicate the study's design with a commonly used term in the title or the abstract	1	"This retrospective cohort study"
		(*b*) Provide in the abstract an informative and balanced summary of what was done and what was found	1	"We analyzed 2722 cases: 751 cervical cancers, 1901 H-SIL. 88 % of patients had never been vaccinated. Among women with H-SIL 85% have never received a vaccination against HPV"
**Introduction**				
Background/rationale	2	Explain the scientific background and rationale for the investigation being reported	3	"Assessing HPV vaccination rates among women with high-grade cervical lesions or cancer allows identification of gaps in prevention, evaluates patterns of vaccine uptake, and informs strategies to enhance coverage and advance elimination initiatives."
Objectives	3	State specific objectives, including any prespecified hypotheses	3	"Our study aimed to determine how many women, with cervical cancer or high-grade dysplasia, were previously vaccinated and how many women had received the vaccine after these diagnoses."
**Methods**				
Study design	4	Present key elements of study design early in the paper	3	"This retrospective observational cohort study"
Setting	5	Describe the setting, locations, and relevant dates, including periods of recruitment, exposure, follow-up, and data collection	3	"conducted between October 15, 2024, and January 31, 2025, by the Health Technology Assessment Committee of Policlinico "G. Rodolico- San Marco", a reference teaching and re-search hospital in Catania, Italy"<br>"We collected information on women residing in the province of Catania, with a population of approximately one million inhabitants, who had a confirmed cytological or histological diagnosis of H-SIL or cervical cancer between January 1, 2003, and December 31, 2020."

Participants	6	(*a*) Give the eligibility criteria, and the sources and methods of selection of participants. Describe methods of follow-up		"met the following criteria: cytological or histological diagnosis of HPV-related cervical cancer or high-grade cervical lesions, residency in the province of Catania during the study period, and presence in the local vaccination registry. Cytological evaluations followed the Bethesda System for Reporting Cervical Cytology [19], while biopsy samples were classified based on the WHO Classification of Tumours, as outlined by the International Agency for Research on Cancer (IARC). Morphology was coded according to the International Classification of Disease for Oncology, third edition"
		(*b*) For matched studies, give matching criteria and number of exposed and unexposed		Not applicable
Variables	7	Clearly define all outcomes, exposures, predictors, potential confounders, and effect modifiers. Give diagnostic criteria, if applicable	3	"For each patient, data were collected on diagnosis date, age at diagnosis, type and classification of the cancer or lesion, and tumour stage at the time of diagnosis."
Data sources/ measurement	8*	For each variable of interest, give sources of data and details of methods of assessment (measurement). Describe comparability of assessment methods if there is more than one group	3	"Information on cervical cancer and high-grade cervical lesions was obtained from the Integrated Cancer Registry of Catania-Messina-Enna"<br>"data on cervical cancer and other cervical lesions were cross-checked between cases registered in the local vaccination registry of the Province of Catania and in the National Register of Causes of Death (RENCAM)"
Bias	9	Describe any efforts to address potential sources of bias		Not applicable
Study size	10	Explain how the study size was arrived at		Not applicable
Quantitative variables	11	Explain how quantitative variables were handled in the analyses. If applicable, describe which groupings were chosen and why	4	"To describe vaccination status distribution, the following parameters were calculated: mean age at diagnosis, number of vaccine doses received at the time of diagnosis, number of vaccines administered post-diagnosis, mean and median time

				between diagnosis and vaccination, vaccination coverage among eligible patients, and the percentage of patients vaccinated after diagnosis."<br>"Vaccination status and age were compared between subgroups based on tumour type, distinguishing between malignant tumours and H-SIL."
Statistical methods	12	(*a*) Describe all statistical methods, including those used to control for confounding	4	"Differences between vaccinated and unvaccinated women were analyzed using Pearson's chi-square test. When expected frequencies were <5 or a cell contained a zero value, statistical significance was verified using the two-tailed Fisher's exact test. In the presence of zero events, the Haldane–Anscombe correction was applied to obtain a stable estimate of the relative risk (RR). A p-value < 0.05 was considered statistically significant."
		(*b*) Describe any methods used to examine subgroups and interactions		"Vaccination status and age were compared between subgroups based on tumour type, distinguishing between malignant tumours and H-SIL."
		(*c*) Explain how missing data were addressed		Not applicable
		(*d*) If applicable, explain how loss to follow-up was addressed		Not applicable
		(*e*) Describe any sensitivity analyses		Not applicable
**Results**				
Participants	13*	(a) Report numbers of individuals at each stage of study—eg numbers potentially eligible, examined for eligibility, confirmed eligible, included in the study, completing follow-up, and analysed	4	"We analysed 2722 cases of women living in the province of Catania, Sicily, Italy diagnosed with cervical tumour or H-SIL between 2003 and 2020"
		(b) Give reasons for non-participation at each stage		Not applicable
		(c) Consider use of a flow diagram		

Descriptive data	14*	(a) Give characteristics of study participants (eg demographic, clinical, social) and information on exposures and potential confounders	5	"The average age of all women at the first vaccination dose was 34,9 years." Table 1
		(b) Indicate number of participants with missing data for each variable of interest		Not applicable
		(c) Summarise follow-up time (eg, average and total amount)		Not applicable
Outcome data	15*	Report numbers of outcome events or summary measures over time	5	"Our study included 2652 women: 751 (28,3%) cervical cancer, 1901 (71,1%) H-SIL. Only 11,9 % had received at least one dose of the vaccine, while 88.1 percent had never been vaccinated" Table 1
Main results	16	(*a*) Give unadjusted estimates and, if applicable, confounder-adjusted estimates and their precision (eg, 95% confidence interval). Make clear which confounders were adjusted for and why they were included	6	Table 1
		(*b*) Report category boundaries when continuous variables were categorized		Not applicable
		(*c*) If relevant, consider translating estimates of relative risk into absolute risk for a meaningful time period		Not applicable
Other analyses	17	Report other analyses done—eg analyses of subgroups and interactions, and sensitivity analyses		Not applicable
**Discussion**				
Key results	18	Summarise key results with reference to study objectives	7	"We found statistically significant differences in the frequency of high-grade squamous intraepithelial lesions and cervical carcinoma between vaccinated and unvaccinated women"
Limitations	19	Discuss limitations of the study, taking into account sources of potential bias or imprecision.	10	"Some vaccinations may not have been captured in our cohort if they were administered in other provinces, potentially leading to underestimation of vaccination

		Discuss both direction and magnitude of any potential bias		rates. We did not record behavioural data from our patients.”
Interpretation	20	Give a cautious overall interpretation of results considering objectives, limitations, multiplicity of analyses, results from similar studies, and other relevant evidence	11	“, among women living in the province of Catania, the limited spread of vaccination caused cases of precancerous lesions or tumours, most of which could probably have been avoid-ed. We found statistically significant differences in the frequency of H-SIL and cervical carcinoma between vaccinated and unvaccinated women. Few cases performed vaccination as secondary prevention, after the appearance of cervical lesions and even less wide-spread is post-conization vaccination as tertiary prevention”
Generalisability	21	Discuss the generalisability (external validity) of the study results		Not applicable
**Other information**				
Funding	22	Give the source of funding and the role of the funders for the present study and, if applicable, for the original study on which the present article is based		Not applicable

*Give information separately for exposed and unexposed groups.

Note: An Explanation and Elaboration article discusses each checklist item and gives methodological background and published examples of transparent reporting. The STROBE checklist is best used in conjunction with this article (freely available on the Web sites of PLoS Medicine at http://www.plosmedicine.org/, Annals of Internal Medicine at http://www.annals.org/, and Epidemiology at http://www.epidem.com/). Information on the STROBE Initiative is available at http://www.strobe-statement.org.
